# The first occurrence of a probable pachycephalosaurian hyoid element

**DOI:** 10.7717/peerj.21719

**Published:** 2026-10-21

**Authors:** D. Cary Woodruff, Holly N. Woodward, Laura Rooney, Joseph Frederickson

**Affiliations:** 1 Phillip and Patricia Frost Museum of Science, Miami, FL, United States; 2 Museum of the Rockies, Bozeman, Montana, United States; 3 American Museum of Natural History, New York, New York, United States; 4 Department of Anatomy and Cell Biology, Oklahoma State University, Tulsa, OK, United States; 5 The Children’s Museum of Indianapolis, Indianapolis, IN, United States

**Keywords:** Pachycephalosaur, Ceratobranchial, Dinosauria, Hyoid

## Abstract

Derived from the second pharyngeal arch, the hyoid complex is a free-floating midline structure that is situated posteroventrally to the lower jaw. While there are many known free-floating osseous structures—such as dermal ossifications, dinosaurian ossified tendons/myosepta, and the mammalian baculum/baubellum—the hyoid complex is one of the few examples of these ‘floating’ bony elements that is found widely across the vertebrate bauplan. In terrestrial vertebrates, the hyoid complex is an important structure for vocalization, feeding, and respiration. A gracile, delicate, and multi-component structure in close proximity to heavily predated tissues, the hyoid complex is not a taphonomically favored structure; and yet, despite these taphonomic challenges, within Dinosauria, the primary osseous portion of the hyoid complex, the ceratobranchial, is sufficiently documented within many clades—save Pachycephalosauria. Previously, there were no known or suspected ceratobranchials reported from any pachycephalosaurian, making it one of the few osseous elements entirely unknown from this clade. Here, we report on a bony element which we hypothesize to be a right ceratobranchial from the holotype of the late Maastrichtian “*Dracorex hogwartsia*.” This element was found associated with the holotypic material—though never reported—and its overall morphology, such as being anteroposteriorly elongate and transversely thin (*e.g*., ‘rod-like’), is congruent with ceratobranchial morphology from other dinosaurian taxa. If our identification is correct, this not only marks the first occurrence, and one of the remaining ‘blank spots’ of the pachycephalosaurian skeleton to be ‘filled in,’ but also has potential physiological and osteohistological implications for better understanding this enigmatic dinosaurian clade.

## Introduction

With their spherically inflated and peripherally ornamented frontoparietal domes, Pachycephalosauria represent some of the most iconic and bizarre of the non-avialan dinosaurs. Constituting a largely fused and hypermineralized mass of bone, which could be upwards of 25 cm dorsoventrally thick (*Pachycephalosaurus*; Woodruff & Ackermans, 2024), the frontoparietal dome (hereafter ‘dome’) of a pachycephalosaurian was an incredibly robust and taphonomically stable portion of the skeleton (Mallon & Evans, 2014); and a majority of pachycephalosaurian taxa are known exclusively from domes and peripheral elements (such as the squamosal). As of 2013, 86% of the North American pachycephalosaurid record was represented by these taphonomically favorable elements (and peripheral dome elements; Evans et al., 2013). While known from approximately 20+ genera (though pachycephalosaurian taxonomy is contentious), to date, only *c*. 30% preserve any postcranial material. Of those specimens with postcrania, most are highly fragmentary and incomplete, and it was not until the 2025 description of the exceptionally complete Early Cretaceous *Zavacephale rinpoche* (Chinzorig et al., 2025) that the first manual elements from any pachycephalosaurian was known. Thus, given their extreme rarity, every postcranial element represents a significant addition to our knowledge of the pachycephalosaurian skeleton.

However, while there are still regions of the pachycephalosaurian skeleton that are in dire need of additional material, and aspects of the skeleton that remain factually unknown (*e.g*., the number of cervical vertebrae), there is an osseous element that remains entirely elusive: the ceratobranchial. Developmentally, the hyoid complex derives from the second pharyngeal arch (Kardong, 2006). In mammals, the hyoid is fused into a single element, while in many other vertebrates, the hyoid is a complex of osseous and cartilaginous components; and in non-avialan dinosaurs, the ceratobranchial is most often the only osseous element within the hyoid complex (Yoshida, Kobayashi & Fiorillo, 2023). In terrestrial vertebrates, the hyoid complex is regarded as an important structure for vocalization (Hilloowala, 1975; Lieberman et al., 2001; Weissengruber et al., 2002), feeding (Lieberman, 2007), and breathing (Van de Graaff et al., 1984). Within the dinosaurian record (and specifically the non-avialan record) ceratobranchials are fairly well documented within clades such as: Abelisauridae (Bonaparte, Novas & Coria, 1990), Allosauridae (Chure & Loewen, 2020), Ankylosauridae (Hill et al., 2015), Brachiosauridae (Janensch, 1935; Chure et al., 2010), Camarasauridae (Gilmore, 1925), Ceratopsia (numerous basal and derived taxa; Morschhauser & Lamanna, 2013), Coelophysidae (Rowe, 1989), Diplodocidae (Tschopp & Mateus, 2017; Woodruff et al., 2018), Dromaeosauridae (Xu, Wang & Wu, 1999; Pei et al., 2014; Lü & Brusatte, 2015; Currie & Evans, 2020), Eusauropoda (Zhang, 1988), Hadrosauridae (Morschhauser & Lamanna, 2013), Heterodontosauridae (Thulborn, 1974), Hypsilophodontidae (Galton, 1974), Iguanodontia (Morschhauser & Lamanna, 2013), Massospondylidae (Li, Zhou & Clarke, 2018), Metriacanthosauridae (Currie & Zhao, 1993), Nodosauridae (Hill et al., 2015), Ornithomimidae (Kobayashi & Lu, 2003), Oviraptorosauria (Xu, Zheng & You, 2010), Stegosauridae (Gilmore, 1914; Morschhauser & Lamanna, 2013), Thescelosauridae (Boyd, 2014), Titanosauria (Zaher et al., 2011; Poropat et al., 2023), Troodontidae (Russell & Dong, 1993), and Tyrannosauridae (Bakker, Williams & Currie, 1988; Brusatte, Carr & Norell, 2012) among others. Herein, we report on what is to our knowledge, the first documented pachycephalosaurian ceratobranchial.

## Materials and Methods

The element in question consists of what we identify as a partial right ceratobranchial. Fragmentary pieces of the left ceratobranchial were also recovered, but do not warrant further discussion other than to say their represented constituents correspond to the more complete contralateral element. The material was collected in association with the holotype of the taxonomically debated Hell Creek Formation “pachycephalosaurini” “*Dracorex hogwartsia*” (The Children’s Museum of Indianapolis (TCMI) 2004.17.1). Interestingly, Bakker et al. (2006) only describes the holotype as consisting of an essentially complete skull, an isolated tooth, and four cervical vertebrae, though additional elements and fragmentary material collected with the holotypic material was accessioned at TCMI at the time of the descriptive analysis. Unfortunately, at this time no quarry maps or excavation photographs are known to exist. However, personal communications on file at TCMI indicate that the specimen described here was discovered approximately “2 feet” (~61 cm) away from the rest of the skull. No other fossil material was reported with the holotypic material, so we do not know if the specimen was isolated, or from a lag deposit. As demonstrated by Arbour et al. (2016) regarding the “furculae” of the taxonomically debated Hell Creek Formation dromaeosaurid “*Dakotaraptor steini*,” chimeric compositions could be constructed from lag deposits and/or misidentification of material. While we cannot attest to the fossiliferous nature of the “*Dracorex*” type locality, given that all of the other material is definitively pachycephalosaurian, in addition to the general rarity of isolated ceratobranchials, at this time we would suggest the most parsimonious explanation is that it indeed belongs to TCMI 2004.17.1.

The specimen was examined and photographed in-person using standard measurement and photographic documentation procedures. While the preservation of osseous constituents of the hyoid apparatus is well documented within Dinosauria, detailed examination of their morphology, anatomical position, and myological associations are severely lacking (but there are notable exceptions such as Morschhauser & Lamanna (2013), Hill et al. (2015), and Yoshida, Kobayashi & Fiorillo (2023)). Even documentation of anatomical planes is lacking, and for descriptive purposes, we primarily relied on the descriptive text and photodocumentation of Ostrom (1961), Sternberg (1951), Prieto-Marquez (2005), and Morschhauser & Lamanna (2013). Ostrom (1961; pp. 111) states that for ceratobranchial orientation, the anterior morphology is “…slightly concave (along the) anterolateral and posteromedial surfaces…,” and tapers posteriorly terminating in a rounded tip; and we relied on these morphologic descriptors to anatomically orient this element. Comparing this specimen *via* a bracketing perspective (Witmer & Thomason, 1995), does little to elucidate anatomical clarity. In crocodilians, the anterior portion of the ceratobranchial is fairly anteroposteriorly straight with a dorsoventrally expanded and rounded anterior terminus; yet the posterior *c*. half is bent nearly 90° dorsal to the anterior long axis, producing an ‘L-shaped’ element (Johnston, 2023). In Aves, the ceratobranchial can be anteroposteriorly straight and ‘rod-like’ (in *Gallus*; Yoshida, Kobayashi & Norell, 2023), to dorsally curved and ‘boomerang-shaped’ (in *Struthio*; Yoshida, Kobayashi & Norell, 2023). However, perhaps the most dramatic hyoid apparatus are in members of Picidae, in which the paraglossal, basihyal, urohyal, ceratobranchial, and epibranchial form a structure that goes posteroventrally from the dentary and dorsoanteriorly wraps up and over the cranium (Jung et al., 2016). Because the ceratobranchial can be curved in multiple planes, is not situated linearly, and can have variable inclination depending on head orientation, for descriptive purposes, the long-axis of the element is situated horizontally, and all directional planes derive from this position.

### Orientation and description

In comparison to ceratobranchials from Aves and crocodilians, as well as known non-avialan dinosaur examples, the anterior region of this element is proportionally dorsoventrally taller than the opposing end. Additionally, the posteriormost region of the ceratobranchial is rounded, which is the articular contact with the epibranchial. Though incomplete, one side of the TCMI 2004.17.1 element is dorsoventrally taller, with an opposite end that has a rounded surface; therefore, given these corresponding morphologies, we have oriented the anterior-posterior axis as such. While more variable, the ceratobranchial of non-avialan dinosaurs appears to typically have a rounded ventral margin with more of a ‘keeled’ dorsal counterpart (compared to more ‘rod-like’ avian ceratobranchials; for non-avialan examples, see Prieto-Marquez (2005) fig. 3 and Hill et al. (2015) fig. 3). The TCMI 2004.17.1 element likewise has one long-axis surface that is rounded, with the opposing being ‘keeled;’ as such, we identify these surfaces as ventral and dorsal (respectively). Additionally, Ostrom (1961) noted that the anterior region of the ceratobranchial has a concave surface along the anterolateral and posteromedial surfaces. Again, though incomplete, as identified, the anterior region of the TCMI 2004.17.1 element has an anteroposteriorly oriented midline concavity (see description below). With a tapered posterior end, ‘keeled’ dorsal and rounded ventral margins, and a dorsoventrally expanded and concave anterior, we anatomically identify this element as the right ceratobranchial. The element is incomplete and measures 121.9 mm in its greatest length anteroposteriorly (Figs. 1A–1E). Laterally, there is a prominent ~midline concavity that is approximately 10 mm anteriorly, tapering to 2.5 mm posteriorly (Fig. 1C). Near the posterior end of this concavity is a small (2.6 mm), anteroposteriorly ovoid neurovascular foramen (Fig. 1D). Compared to the medial side, there are more ridges present laterally. There is one ridge that forms the dorsal margin, and though incomplete, it is 63.3 mm anteroposteriorly long. Along the ventral border, there is another large ridge that is 69.1 mm long. Within the posteriormost *c*. 52 mm, there are several additional dorsal and ventral ridges of varying lengths. Posteriorly, most of these ridges converge towards the midline, and while a slight ‘chevroned’ fiber texture is observed anteriorly, along these posterior ridges the texture orientation changes to nearly entirely inclined. A rugose texture is present on the lateral edge of the expanded posterior terminus, and there is a slightly curved postero-midline ridge from both the anterodorsal and anteroventral peaked margins.

**Figure 1:**
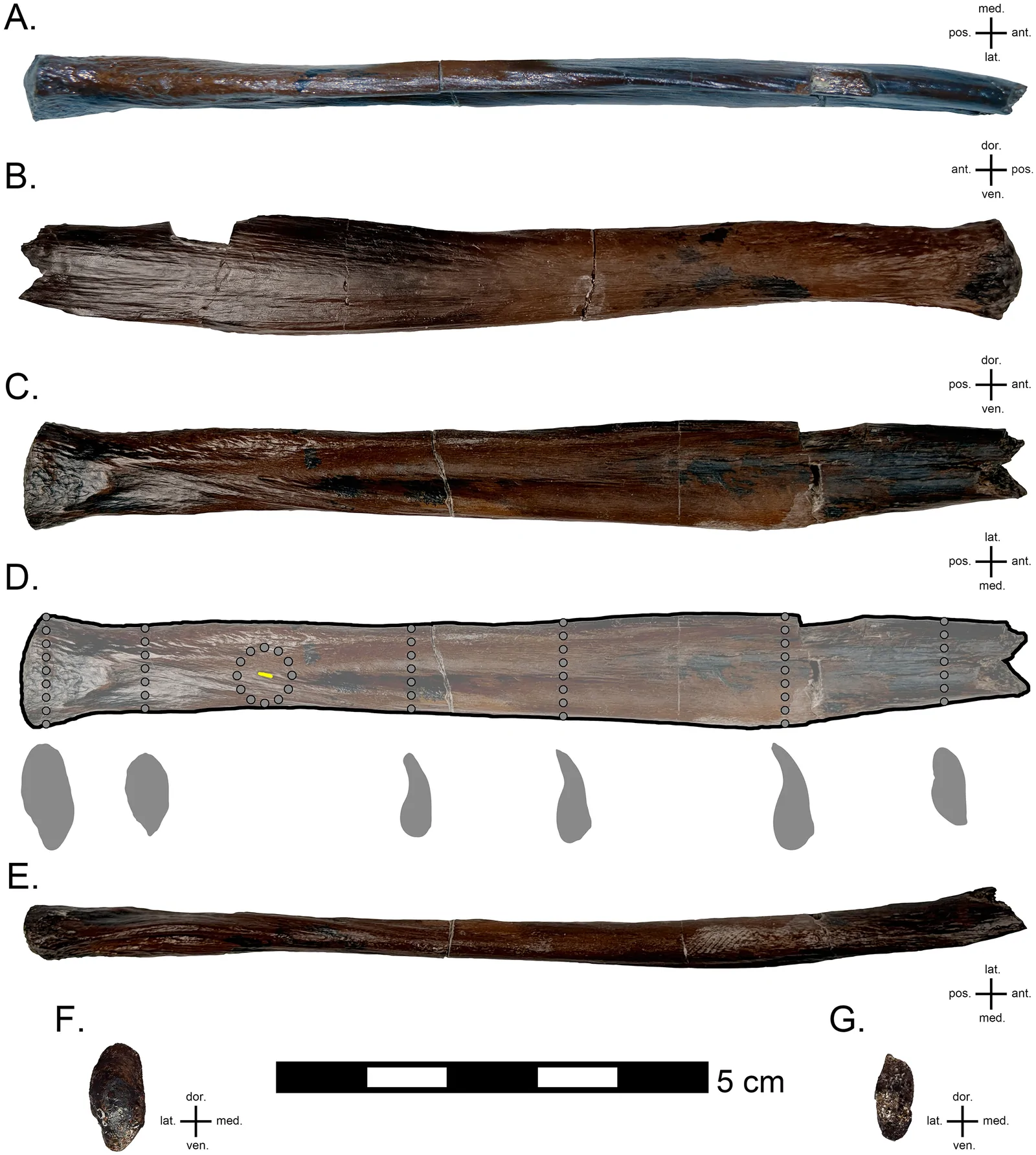
The proposed incomplete right ceratobranchial of “*Dracorex hogwartsia*” (TCMI 2004.17.1). In dorsal (A), medial (B), lateral (C), ventral (E), posterior (F), and anterior (G) views (with respective orientation guides), (D) lateral view of (C) with dotted grey lines indicating the locations of the cross-sectional profiles in grey below. Small yellow oval within the grey-dotted circle denotes the size and location of the neurovascular foramen. ant., anterior; dor., dorsal; lat., lateral; med., medial; ven., ventral. Scale bar = 5 cm.

In medial view (Fig. 1B), the element is fairly straight with a general dorsoventral height of *c*. 11.4 mm. The anterior aspect is missing, and approximately 30 mm before the rounded posterior terminus, there is a 15.1 mm dorsoventrally expanded region. The posterior end has a slight spherical expansion with notable anterodorsal/anteroventral peaked dorsal and ventral margins, and this whole region has a rugose surface texture. Overall, there is a slight convexity, and near the posteroventral margin is a 28.9 mm anteroposteriorly long ridge (likely a muscle attachment site), and approximately 88% of the lateral surface—particularly the anterior region—has a posteriorly oriented ‘chevroned’ fibrous texture.

In anterior view (Fig. 1G), though broken, the ventral edge is rounded, and there is a slight medially oriented flat surface posteroventrally. Transversely, the ventral region is the widest, being 5.68 mm thick, quickly dorsally tapering to 2.2 mm thick. In cross-section, the rounded ventral profile, the tapered dorsal margin, the medial convexity, and the lateral concavity produce an anterior cross-sectional profile shaped like an inverted comma (Fig. 1D). The posterior region of this element is complete, and given its slight dorsoventral and transverse expansion, along with its rounded terminus (Figs. 1A–1F), appears essentially identical to the articular surface for the epibranchial in extant archosaurs. In posterior view (Fig. 1F), the cross-sectional shape is somewhat ‘D’-shaped—the lateral edge is dorsoventrally straight and curved medially. The ventrolateral edge is angled medioventrally, and there is a corresponding groove present transversely across from this angled surface on the medial side. Dorsoventrally, the posterior face is 14.5 mm tall, and it is 7.2 mm thick at its transversely widest portion.

In dorsal view (Fig. 1A), the element is very anteroposteriorly straight. The transversely widest portion of the anteriormost end is 6.2 mm thick, and most of the element is uniform in transverse thickness (*c*. 4.3 mm). Along the length of the element, there are two slight transverse bulges: the first is posterior to the broken anterior end, which is 5.8 mm thick, and the second is near the rounded posterior terminus and is 5.5 mm thick. The prominently peaked dorsal edge is present along most of the length of the element. In ventral view (Fig. 1E), the morphology is fairly uniform—a rounded edge is present along most of the surface, save for a ridge along the posteriormost 31 mm. As observed in dorsal view, the element is uniform in transverse thickness, save the two slightly expanded regions.

## Discussion

### Possible alternative identifications

Assuming that this element is indeed pachycephalosaurian, if not a ceratobranchial, what are other possible candidates? Given that TCMI 2004.17.1 also preserved four cervical vertebrae (Bakker et al., 2006), it is reasonable to suspect that this element could instead represent a cervical rib. Unfortunately, no pachycephalosaurian cervical ribs are yet documented in the literature, but general morphologic comparisons to other dinosaurian cervical ribs are possible. At least in terms of post-atlas cervical ribs, these elements can indeed be anteroposteriorly elongate and have convex/concave lateral/medial surfaces—like the overall morphology of this element from TCMI 2004.17.1; but this is where any similarities end. In cross-sectional view, though variable along the length, a cervical rib has a cross-sectional profile ranging anteriorly from ‘crescent’ to ‘kidney bean’-shaped, to posteriorly tapering and becoming more of a dorsoventrally elongate oval. The TCMI 2004.17.1 element is more consistent in dorsoventral thickness and does not posteriorly taper to a point as a cervical rib would. While portions of a cervical rib have the ‘crescent’ cross-sectional profile, that shape is restricted to portions of its length, whereas in the TCMI 2004.17.1 element, this cross-sectional profile is present for most of the length. The posterior end of a cervical rib tapers to a point, but in the TCMI 2004.17.1 element, the posterior end is expanded with a rounded posterior face. Additionally, cervical ribs do have a fibrous texture, but such is generally anteroposteriorly oriented, not chevroned as in the TCMI 2004.17.1 element. Finally, while cervical ribs can indeed be anteroposteriorly longer than their respective centra, if this element was alternatively a cervical rib that corresponded to cervical vertebra “3,” “8,” or “9” from TCMI 2004.17.1 (Bakker et al., 2006), then it would be over 3× anteroposteriorly longer than the centra—a centrum:cervical rib ratio more similar to mamenchisaurid sauropods.

While this element from TCMI 2004.17.1 does not resemble most cervical ribs, we must note the comparison to the atlantal rib. The atlas (cervical vertebra 1) is morphologically the least complex vertebra in the cervical series, and it too possesses paired articulating ribs. Within non-avialan dinosaurs, unlike the posteriorly proceeding elements, the atlantal rib is unicapitate, and in general overview, the atlantal rib is anteroposteriorly straight, transversely thin, and has a rounded end that articulates with the parapophysis (*e.g*., ‘splint’-like; Fig. 2). Superficially, this morphology is more consistent with that of the element described here, and TCMI 2004.17.1 does preserve the atlas. As with ceratobranchials, more detailed descriptive text dedicated to the atlantal rib is needed. In a review of the morphology and evolution of the atlantal rib in *Archaeopteryx* sp., Tsuihiji (2017) shows the degree of variability within several non-avialan dinosaurs. For example, in *Tenontosaurus tilleti*, the atlantal rib is anteroposteriorly straight, sub-uniform in dorsoventral thickness, with dorsoventrally rounded ends—*e.g*., ‘popsicle stick’-like (Fig. 2A). Whereas in *Camarasaurus* cf. *grandis*, the atlantal rib bifurcates with a short dorsoposterior projection, and an elongate posterior portion that tapers to a point (Fig. 2C). While in *Coelophysis kayentakatae*, the entire element is extremely anteroposteriorly elongate, dorsoventrally thin, and tapers gradually along its entire length (Fig. 2B). In *Changchunsaurus parvus*, the atlantal rib is morphologically similar to *T*. *tilleti*, but the posteriormost *c*. third tapers to a point (Fig. 2E). In several of these taxa, the head of the rib can be offset from the long-axis of the element, and the rib head is very spherical. In describing the atlantal rib of *Hypsilophodon foxii*, Galton (1974) states that the body of the element is oval in cross-section. In ventral view, the atlantal rib of *Struthiomimus altus* (Tsuihiji, 2017) and *Stegosaurus stenops* (Gilmore, 1914) is somewhat triangular in profile as it is transversely wide anteriorly and tapers posteriorly. Holland (1906) figures the atlantal rib perhaps more than any other. Though he identifies it as the “supposed rib of the atlas of *Diplodocus*” (Holland, 1906; fig. 20 pp. 249; reidentified as *Galeamopus hayi* (Tschopp, Mateus & Benson, 2015); Fig. 2F), he figures this element in lateral, medial, and ventral views. While the entire element is generally anteroposteriorly straight, it is dorsoventrally tallest anteriorly, and tapers posteriorly. Dorsally, there are two prominent processes, and both dorsal and ventrally, the edge appears keeled. In ventral view, the larger of the two dorsal processes is inclined medially; and a medially inclined dorsal projection was also illustrated by Bonaparte, Novas & Coria (1990) for the atlantal rib of *Carnotaurus sastrei*. While no two of these aforementioned atlantal ribs are morphologically identical, in addition to the spherically inflated rib head, they all appear to possess a straight or slightly undulating anteroposteriorly oriented keel laterally.

**Figure 2:**
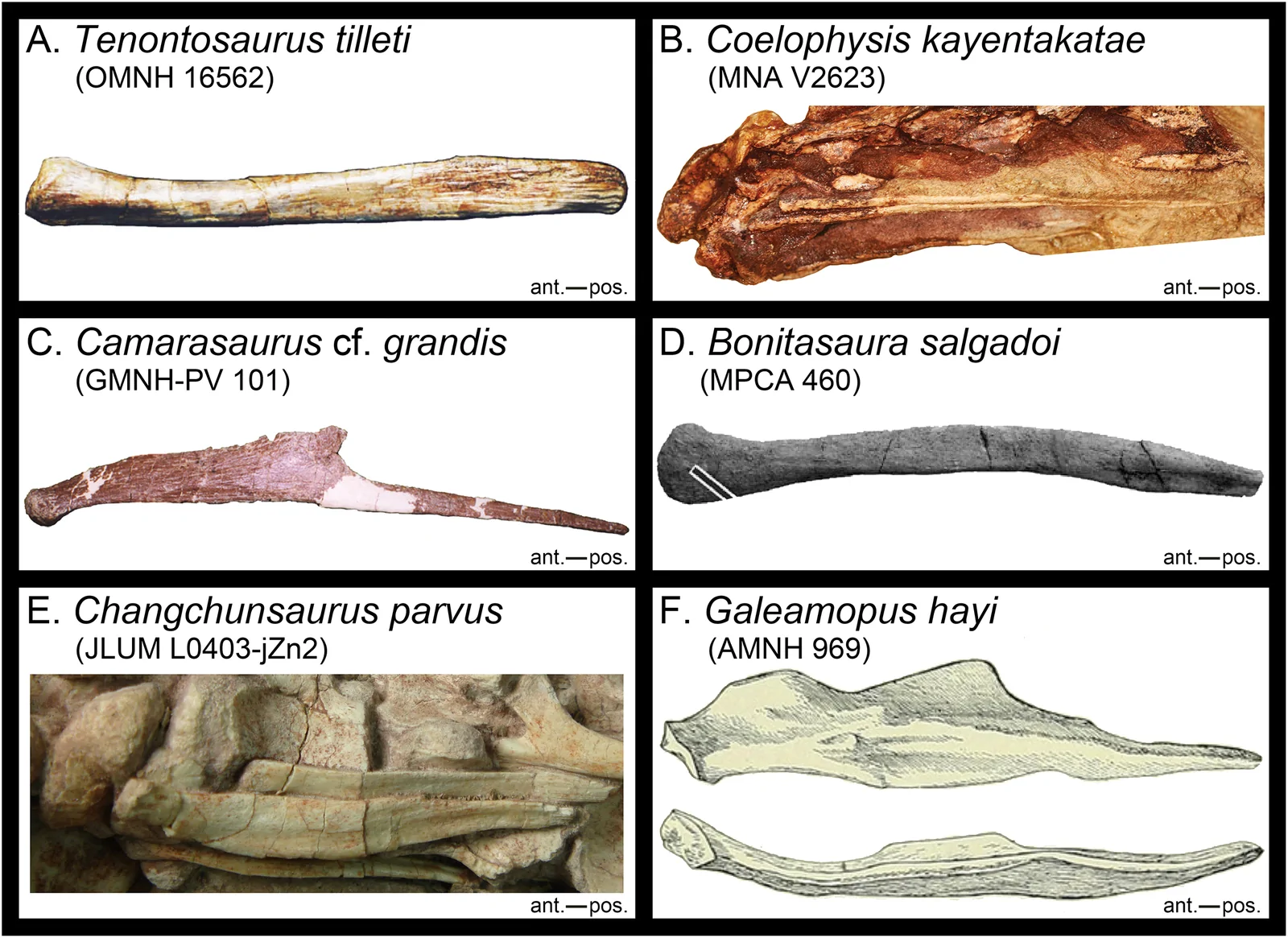
Comparisons between non-avialan dinosaur atlantal ribs. (A) *Tenontosaurus tilleti* (OMNH 58340; from Tsuihiji (2017)). (B) *Coelophysis kayentakatae* (MNA V2623; from Tsuihiji (2017)). (C) *Camarasaurus* cf. *grandis* (GMNH-PV 101; from Tsuihiji (2017)). (D) *Bonitasaura salgadoi* (MPCA 460; from Gallina & Apesteguía (2015)). (E) *Changchunsaurus parvus* (JLUM L0403-jZn2; reflected). Photograph provided by R. Butler and C. Jun. (F) *Galeamopus hayi* (AMNH 969; top orientation lateral, bottom ventral; from Holland (1906)). Not to scale. *ant., anterior; pos., posterior*.

Again, while the TCMI 2004.17.1 element shares several similarities to these atlantal ribs, these congruent morphologies are more likely due to the overall ‘simple’ nature of these respective elements. The TCMI 2004.17.1 element does not posteriorly taper as in most atlantal ribs; it is not oval in cross-section, nor does it have the medially inclined dorsal process; and it does not have the lateral midline keel, but instead a midline concavity. Most notably, presuming our orientation for the TCMI 2004.17.1 element is correct, while there is a rounded end, it is dorsoventrally ovoid rather than spherical, and it is located on the posterior end rather than the anterior; both morphologies that are contrary to the condition that would be expected of an atlantal rib. In short, while the only other possible initial element identification could be a cervical rib, and of those, the atlantal rib being the best candidate, there are numerous morphologic differences that preclude such. As such, our identification of this element representing a ceratobranchial remains.

### Comparisons to known dinosaurian ceratobranchials

While ceratobranchials are often more simplistically described as “rod-like,” while not an erroneous generality, there is contrarily quite more morphologic variability than what is largely portrayed in the literature (Fig. 3). For example, in the hadrosaurid *Corythosaurus casuarius* (American Museum of Natural History (AMNH) 5388), Ostrom (1961) illustrates the ceratobranchial as gently sigmoidal along a horizontal plane, with a tapered and dorsally curved posterior end, compared to an extremely dorsoventrally flared anteriormost region. Conversely, in *Brachylophosaurus canadensis* (Museum of the Rockies (MOR) 794 and MOR 1071), Prieto-Marquez (2005) shows the photographed ceratobranchial, while exhibiting a tapered posterior end and a dorsoventrally flared anterior end, is nearly uniformly ventrally curved, with a shallow ‘U’-shaped profile (Fig. 3B). Perhaps these differences are due to taxonomic variation, variation between lambeosaurines and saurolophines, functional differences in the hyomandibular apparatus in these taxa, and/or some combination thereof. In the ankylosaurid *Pinacosaurus grangeri* (Institute of Geology, Ulaan Baatar, Mongolia (IGM) 100/3186; Fig. 3F), Hill et al. (2015) showed the ceratobranchials with fairly uniform anterior/posterior ends and laterally having a wide ‘U’-shape. In various specimens from multiple taxa, these hyoid elements have been left articulated/associated with the skull, so exact multi-planar morphologic comparisons are not possible. For example, in the *Saurornitholestes langstoni* (University of Alberta, Laboratory of Vertebrate Palaeontology (UALVP) 55700) described by Currie & Evans (2020), the lateral aspects of the ceratobranchials are not readily viewable, but ventrally and ventrally oblique, these elements appear essentially as anteroposteriorly straight as that of TCMI 2004.17.1. In *Nanotyrannus lancensis* (Cleveland Museum of Natural History (CMNH) 7541; Fig. 3A), in lateral view, the ceratobranchial has a gentle anteroposterior ‘U’-shape, somewhat uniform anterior/posterior ends—but a much more dorsoventrally thickened mid region, and in ventral view, a notable laterally oriented curvature. Meanwhile, in the diplodocine *Galeamopus pabsti* (Naturmuseum Zürich (NMZ) 1000011; Tschopp & Mateus, 2017), lateromedially, the ceratobranchial is fairly uniform dorsoventrally, and very ‘U’-shaped with a strong dorsally oriented curvature.

**Figure 3:**
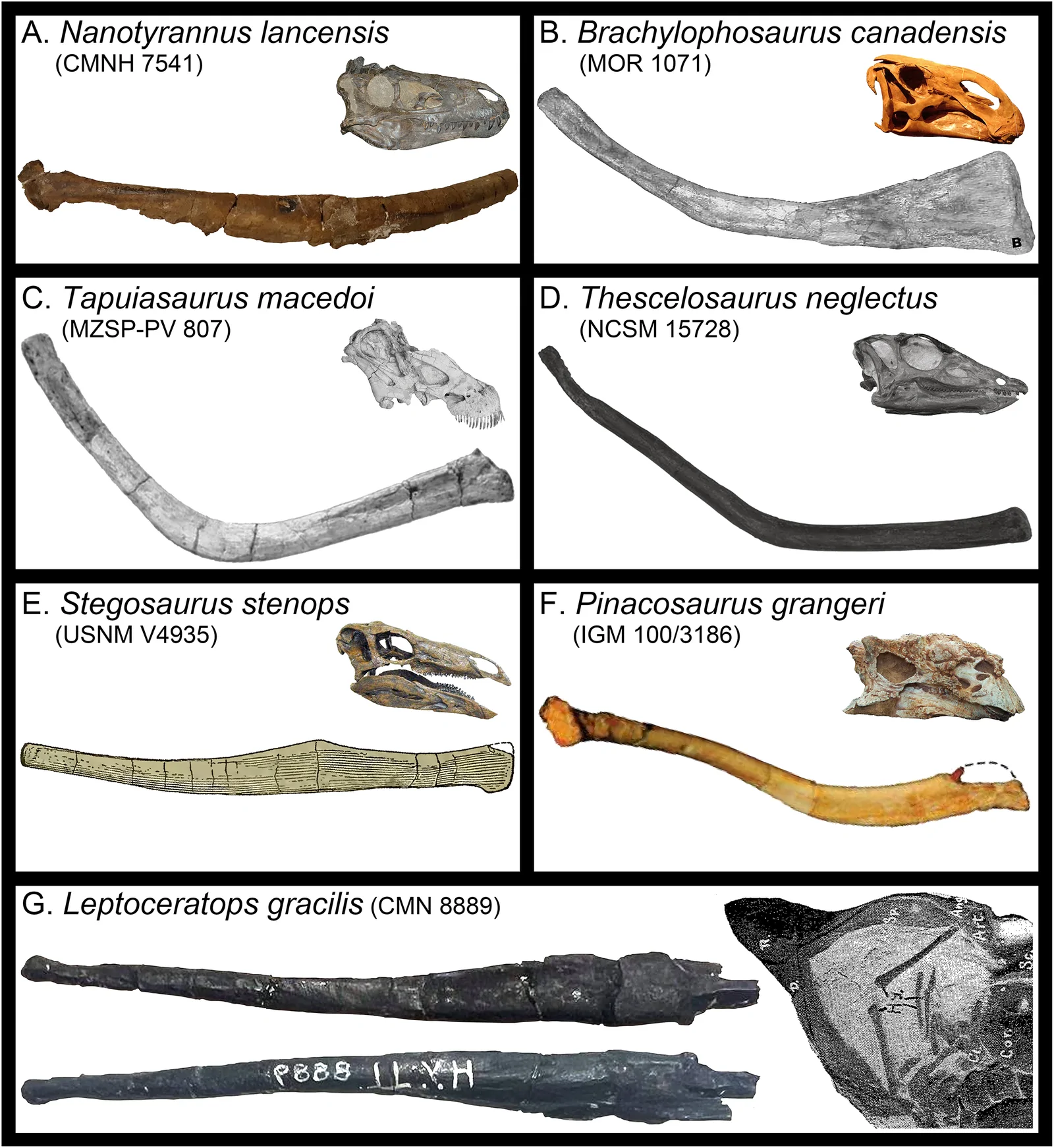
Comparisons between non-avialan dinosaur ceratobranchials. (A) *Nanotyrannus lancensis* (CMNH 7541). Photographs by the Cleveland Museum of Natural History. (B) *Brachylophosaurus canadensis* (MOR 1071). Photograph of ceratobranchial from Prieto-Marquez (2005). Photograph of MOR 794 skull by Museum of the Rockies. (C) *Tapuiasaurus macedoi* (MZSP-PV 807). Photographs from Zaher et al. (2011). (D) *Thescelosaurus neglectus* (NCSM 15728). Photographs from Boyd (2014). (E) *Stegosaurus stenops* (USNM V4935). Illustration from Gilmore (1914). (F) *Pinacosaurus grangeri* (IGM 100/3186). Photograph from Hill et al. (2015). (G) *Leptoceratops gracilis* (CMN 8889). The more complete of the two ceratobranchials is shown here reflected. Photographs provided by J. Mallon. Image on the right is the articulated specimen of CMN 8889 in ventral view with the articulated/associated hyoid elements (from Sternberg (1951)). Not to scale. ant., anterior; pos., posterior.

Within known non-avialan dinosaur ceratobranchials, those from ceratopsians are morphologically very similar to that of TCMI 2004.17.1 (Marginocephalia = Ceratopsia + Pachycephalosauria), and particularly so to *Leptoceratops gracilis* (CMN 8889; Fig. 3G). The two ceratobranchials are not complete, but it would appear that this element has a slightly dorsoventrally flared and angled anterior region, and tapers posteriorly. Notably, the rest of the element is largely anteroposteriorly and lateromedially straight—as in the TCMI 2004.17.1 element. However, the TCMI 2004.17.1 element is not as posteriorly tapered as that of CMN 8889. Additionally, while the external surface of CMN 8889 appears fragmented (and possibly painted), there is what appears to be a posteriorly oriented ‘chevroned’ texture as in the TCMI 2004.17.1 element. Perhaps the most interesting, articulated immediately anterior to the ceratobranchials of CMN 8889 is a larger pair of anteromedially inclined elements that (Sternberg, 1951) identified as the “first ceratobranchials.” Yet in regard to the elements discussed above, Sternberg (1951; p. 242) wrote: “I have no idea what they could be, unless they represent part of the hyoid arch, and if they do, one would expect them to represent the second ceratobranchials.” Of note, while Morschhauser & Lamanna (2013) discuss these ‘secondary ceratobranchials’ and Sternberg (1951) assessment, they note these elements may more likely represent ossified ceratohyals (although their fig. 2 identifies them as “probable first ceratobranchials”). Yang (2025) additionally noted that some hyoid elements in non-avialan dinosaurs have been previously misidentified—leading to subsequent identification and developmental/functional confusion. Given the paucity of information surrounding archosaurian hyomandibular osteology, we will not attempt to identify the TCMI 2004.17.1 element as either ‘ceratobranchial I’ or ‘ceratobranchial II,’ nor would we alternatively identify it as any other hyobranchial component; though we welcome alternative identifications as knowledge of this overlooked region increases.

### Function of the pachycephalosaurian ceratobranchial

Li, Zhou & Clarke (2018) noted that elongation of the ceratobranchials (and other hyobranchial elements) is associated with tongue movement and mobility. As elongation increases, there is also an increase in the ossification of the other hyobranchial elements; all of which are associated with increasing function of the tongue. Li, Zhou & Clarke (2018) hypothesized that such increases within Archosauria corresponded to the tongue’s mobility and its role towards intraoral processing, diet, and the use of forelimbs in food manipulation.

Though the TCMI 2004.17.1 element is incomplete, the ceratobranchial is approximately 30% the anteroposterior length of the skull (Fig. 4B). In review of other non-avialan dinosaur ceratobranchial:skull ratios, this can range from *c*. 25% in *Massospondylus carinatus*, *c*. 26% in *Linheraptor exquisitus*, *c*. 30% in *Saurornitholestes langstoni*, *c*. 35% in *Brachylophosaurus canadensis*, *c*. 41% in *Nanotyrannus lancensis* and *Thescelosaurus neglectus*, to 50% in *Camarasaurus lentus* (Fig. 4B). To reiterate, the entire archosaurian hyobranchial apparatus is in dire need of study, but if these rudimentary and preliminary ratios signify an accurate trend, they could be supportive of the hypotheses of Li, Zhou & Clarke (2018). While pachycephalosaurians have always been depicted with short forelimbs, it was not until *Zavacephale rinpoche* (Chinzorig et al., 2025) that we could factually quantify such reconstructions, and with such short forelimbs, they likely functioned less in intraoral food manipulation. In taxa with longer forelimbs, such as *M*. *carinatus*, the ceratobranchial:skull ratio is smaller, and *vice versa* (*e.g*., *N*. *lancensis*)—*sensu* Li, Zhou & Clarke (2018; Fig. 4A). Potentially, in taxa with the greatest ceratobranchial:skull ratios, such as *C*. *lentus*, even though the forelimb was greatly elongated, the manus could not function in food manipulation, hence the greater length of the ceratobranchial (Fig. 4A). Therefore, given that the ceratobranchial of TCMI 2004.17.1 was >30%, and that this taxon likely possessed greatly reduced forelimbs, we could tentatively postulate that pachycephalosaurians may have relied on a more mobile tongue for intraoral food manipulation. An additional way to examine this hypothesis may be *via* the surface textures across this element. Though it is outside the scope of this current analysis given that these varied textures are (i) well preserved, (ii) present across the element, and (iii) likely constitute soft tissue attachment sites, perhaps myology could be reconstructed, and from there, the role/significance of these muscles in feeding as well (Li & Clarke, 2015).

**Figure 4:**
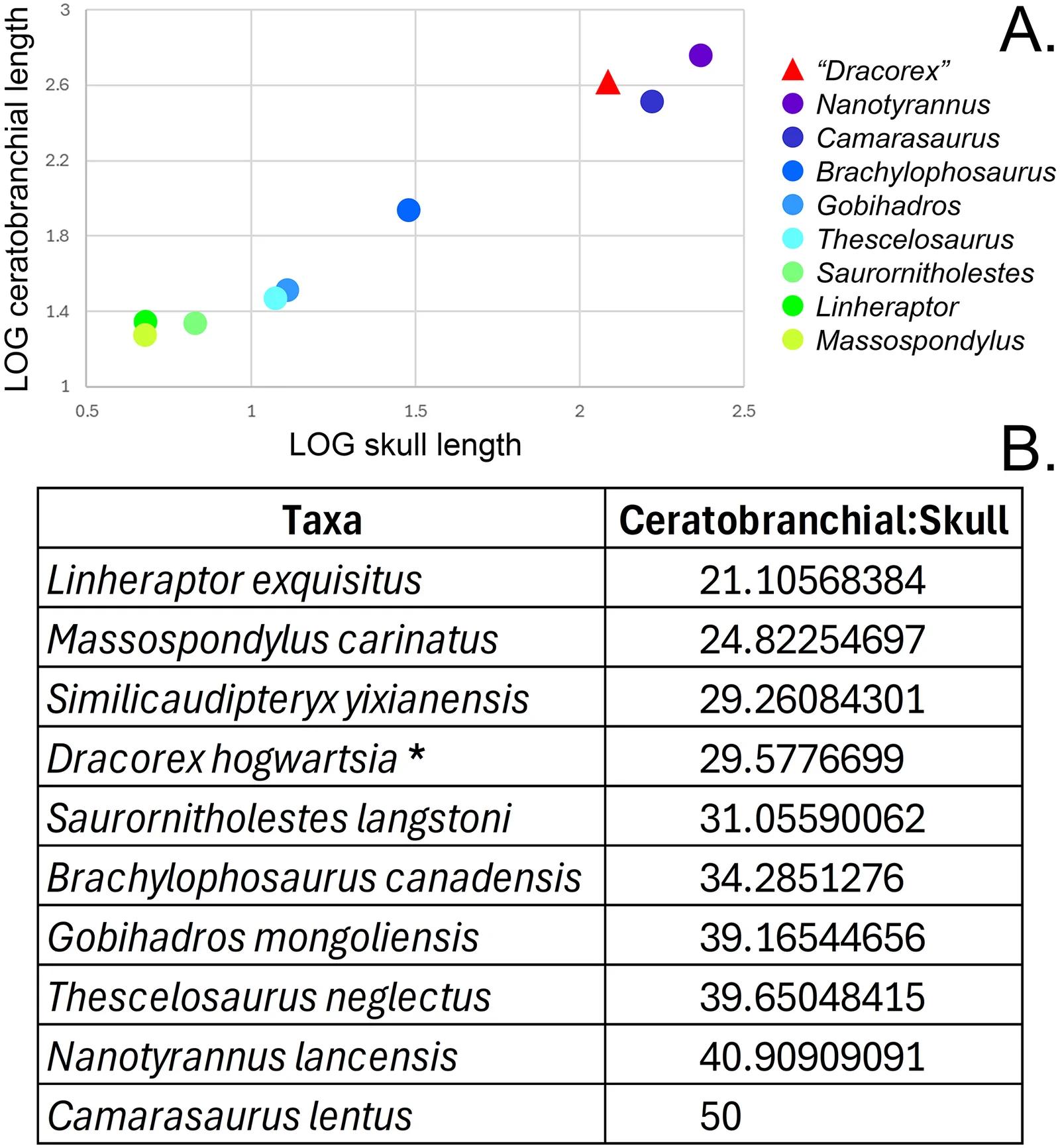
Ceratobranchial to skull length analyses. (A) LOG skull length *vs*. LOG ceratobranchial length. “*Dracorex*” denoted with red triangle; key to right of graph. Note how taxa such as “*Dracorex*,” *Nanotyrannus*, and *Camarasaurus*—those with a manus that could not function in food manipulation have a larger ceratobranchial relative to skull length. (B) Percentage value of ceratobranchial to skull length. *Indicates that the ceratobranchial of “*Dracorex*” is incomplete. As in (A), note how taxa with manus/forelimb incapable of food manipulation have a larger ceratobranchial in relation to skull length.

### Implications for life history analyses

TCMI 2004.17.1 represents a catalyst and controversial specimen towards our understanding of pachycephalosaurian paleobiology. Was *Dracorex hogwartsia* a valid and coeval taxon alongside *Pachycephalosaurus wyomingensis* and *Stygimoloch spinifer* (Bakker et al., 2006)? Or, alternatively, do the ‘flat-headed’ but large squamosal horns of *D*. *hogwartsia* indicate that it was an ontogimorph of *S*. *spinifer*, and that the Hell Creek Formation still harbored two coeval taxa (*S*. *spinifer* and *P*. *wyomingensis*; Longrich, Sankey & Tanke, 2010)? Or, do the seemingly ‘intermediate’ and ‘transitional’ morphologies between the smallest to largest taxa (*D*. *hogwartsia* → *S*. *spinifer* → *P*. *wyomingensis*) indicate that all three constitute a single ontogenetic lineage (Horner & Goodwin, 2009)? Limb bones are the only elements for which a relationship between body size and ontogenetic status has been extensively validated. The lack of postcranial material conducive towards life history assessments (*e.g*., femora or tibiae) has therefore precluded osteohistological examination of the holotype’s ontogenetic status to date. The holotypic skull of *Nanotyrannus lancensis* (CMNH 7541) likewise presents a similar challenge to that of TCMI 2004.17.1. Originally, both were recognized as novel taxa—only to be embroiled in taxonomic debates pertaining to ontogeny, and both lack traditional skeletochronologically informative postcrania; yet they preserve ceratobranchials. While the analysis of Zanno & Napoli (2025) demonstrated support for two Hell Creek Formation eutyrannosaurs (*via* hindlimb age-determinate osteohistology), the recent study of Griffin et al. (2025) osteohistologically examined the right ceratobranchial of CMNH 7541 and concluded that this element not only recorded and preserved pertinent life history information, but that the ceratobranchial is a viable element for such analyses.

However, an eLetter response (see Woodward et al., 2026 in Griffin et al., 2025) (HB1.1) called attention to problematic methodology and insufficient support for this conclusion. Griffin et al. (2025) identified osteohistological features of the ceratobranchial they consider as indicating cessation of organismal growth, yet acknowledged that the CMNH 7541 ceratobranchial was still elongating posteriorly. Additionally, Griffin et al. (2025) interpreted narrow growth-mark spacing as reflecting asymptotic size, but do not consider that cortical drift can produce the same morphology. Yet most importantly, Griffin et al. (2025) failed to validate the utility of the ceratobranchials for establishing skeletal maturity using an established proxy, as the only ceratobranchial-femur histological comparison in their dataset was in an immature alligator (*Alligator mississippiensis*). To reiterate, despite our concerns over the interpretation surrounding ceratobranchial osteohistology, this does not counter nor question the results of Zanno & Napoli (2025). Much has been interpreted and debated regarding the maturity of TCMI 2004.17.1, and we merely caution against further contention until more absolute osteohistological examinations and ground truthing have been conducted. Therefore, for the interim, we elect to defer consumptive analysis of the TCMI 2004.17.1 ceratobranchial. However, if subsequent examinations support the interpretations of Griffin et al. (2025) and reveal that the ceratobranchial is indeed a skeletochronologically and maturationally informative element, then we copiously agree that sampling of the TCMI 2004.17.1 ceratobranchial should be conducted.

## Conclusions

Herein, we describe a previously unreported element associated from the holotype of “*Dracorex hogwartsia*” that we interpret to represent a partial ceratobranchial—the first such element ever reported from any pachycephalosaurian. The overall morphology is consistent with other known non-avialan dinosaur ceratobranchials, but we note that this is a more morphologically diverse element than generally portrayed. Additionally, we reiterate that archosaurian hyomandibular (and atlantal rib) anatomy is in dire need of in-depth examination and documentation. Finally, the proportional size of this proposed ceratobranchial suggests that pachycephalosaurians may have possessed more mobile tongues, and if so, this may have contributed towards intraoral food manipulation.

## Institutional abbreviations

AMNH: American Museum of Natural History, New York, NY, USA
CMN: Canadian Museum of Nature, Ottawa, Ontario, Canada
CMNH: Cleveland Museum of Natural History, Cleveland, OH, USA
GMNH: Gunma Museum of Natural History, Tomioka, Gunma, Japan
IGM: Institute of Geology, Ulaan Baatar, Mongolia
MPCA: Museo Provincial ‘Carlos Ameghino,’ Cipolletti, Argentina
MNA: Museum of Northern Arizona, Flagstaff, AZ, USA
MOR: Museum of the Rockies, Bozeman, Montana, Canada
NMZ: Naturmuseum Zürich, Zürich, Switzerland
OMNH: Sam Noble Oklahoma Museum of Natural History, University of Oklahoma, Norman, OK, USA
TCMI: The Children’s Museum of Indianapolis, Indianapolis, IN, USA
UALVP: University of Alberta, Laboratory of Vertebrate Palaeontology, Edmonton, Alberta, Canada

## Additional Information and Declarations

### Data Availability

The following information was supplied regarding data availability:

We did not use any code. The specimen that was examined for this study is accessioned at the Children’s Museum of Indianapolis, and is available for scientific examination by all qualified and authorized individuals. To request access to the specimen, contact The Children’s Museum of Indianapolis’ Lead Paleontologist and Manager of Natural Science Collections, Joseph Frederickson, and Curator of Paleontology, Laura Rooney.
